# Anne M. Donnellan (1943–2024): a tribute to an autism legend, mentor, and friend

**DOI:** 10.3389/fnint.2024.1469778

**Published:** 2024-10-22

**Authors:** Steven K. Kapp

**Affiliations:** School of Psychology, Sport, and Health Sciences, Faculty of Science and Health, University of Portsmouth, Portsmouth, United Kingdom

**Keywords:** autism, sensory and movement differences, communication, relationships, least dangerous assumption, presume competence, inclusion, neurodiversity

Anne Donnellan was a powerful advocate, researcher, educator, mentor, and friend to many, a pioneer for the rights and dignity of autistic people and others with disabilities. She started one of the first preschool programs for autistic children when the field tended to dismiss us as unable and unmotivated to learn and relate to people, publishing a guide on how to develop such programs (Donnellan-Walsh et al., 1977). While trained in behavioral psychology and initially promoting positive approaches to managing aggressive and self-injurious behaviors, she became increasingly alarmed about the potential harms of applied behavioral analysis (ABA). This led to an influential book against aversives called *Progress without Punishment* (LaVigna and Donnellan, 1986; Donnellan et al., 1988). She championed inclusion both in the classroom and the community, actively supporting deinstitutionalization (including successfully advocating for our friend Joaquin Carson: Carson, 2017). From her early days as a practitioner, Anne (Donnellan, n.d.) became acquainted with all the major advocates and writers in the field. She entered and advanced in academia to become a Professor at the University of Wisconsin-Madison. She exercised her privilege, for example, by co-editing the influential, original *Handbook of Autism* (Cohen et al., 1987). Ever the progressive thinker with a strong voice, she rocked the establishment. This led to some professional fallout with former collaborators, but she continued to rise, later becoming Provost of the University of San Diego and Director of its Autism Institute. A private Catholic university with a mission to foster ethical and compassionate leaders, the University of San Diego appealed to Anne—a child of Irish Catholic immigrants—because it kept her grounded with loved ones (her son John Walsh and his family lived nearby) while nurturing her feisty appetite for making life better for people (Wisconsin State Journal, 2024).

Frustrated with the use of restraints through behavior (including punishments and seclusion) and chemicals (overmedication), Anne became an advocate of augmentative and alternative communication (AAC). She valued that behavior can be a form of communication (Donnellan et al., 1984) but also that autistic people may struggle to control their bodies and need support (Donnellan et al., 1992). For example, with the support of quality relationships (Donnellan et al., 1992; Robledo and Donnellan, 2008, 2016), autistic people may manage their stress to initiate action (Donnellan et al., 2006).

Anne wrote about and discussed invented knowledge (Donnellan, 1999), pairing it with her critical idea of making the least dangerous (and most helpful) assumption so that the person has the most likely chance of functioning as well as they can (Donnellan, 1984). This means presuming competence (especially but not only about non-speaking people), and empowering people with the opportunity to relate and grow that patronizing interpretations might deny. Anne relatedly distinguished between the ability to speak and the ability to think, and actively opposed dehumanization, isolation or seclusion, and abuse. She valued the self-advocacy of autistic people and understood the true meaning of reciprocity, the role of communication partners in supportive relationships: working with people, not on them (as she put it: USD News Center, 2008). Together with Anne's work with Martha Leary on movement differences (Donnellan and Leary, 1995), Anne's body of work helped to explain how some autistic people may be unable to speak but can communicate in other ways (such as through language and typing).

Anne highlighted how the neurodiversity movement most urgently applies to non-speaking people and autistic people with high support needs including those who may have intellectual disabilities. I first encountered Anne's research efforts in 2007 shortly after learning about the neurodiversity and disability rights movements, through my research for a paper for an undergraduate anthropology class on Native Americans in American public life. Anne's study, led by her former PhD student Jeanne Connors, of people with developmental disabilities in Navajo society (Connors and Donnellan, 1993, 1998) greatly inspired my argument that their traditional inclusive, pragmatic approach may fare better for autistic people than Western society's medical model (this later developed into my first published paper: Kapp, 2011). From 2008 to 2013, I attended and was pleasantly surprised to present at Anne's autism conference, which she co-organized with her former PhD student Jodi Robledo; Anne respectfully welcomed speakers from ‘behind the scenes' at the conference and into her home. Anne's simple act of inviting me to be on the panel in 2008 marked my first academic presentation and felt authentic. Anne invited autistic and parent bloggers from the progressive Autism Hub along with leading presenters on the various aforementioned topics and beyond to combine important concepts with practical applications.

Twenty years after co-founding the first social justice organization for all autistic people (AutCom), Anne (with Martha Leary and David Hill) published her first main theoretical paper on the sensory-movement account of autism in 2010. This paper represented the only psychological or biological theory in the first special issue on autism and neurodiversity, primarily featuring leading activists, and scholars (Savarese and Savarese, 2010). Soon after in 2012 Anne and Martha published their second book on this topic (Leary and Donnellan, 2012). In 2013 she co-edited (Torres and Donnellan, 2015b) a Research Topic on Autism: The Movement Perspective (Torres and Donnellan, 2015a) in *Frontiers in Integrative Neuroscience* with Liz Torres. This special issue included her important contributions on theory (Donnellan et al., 2013) and perspectives (Robledo et al., 2012) of autistic people. This account encompassed not only motor and sensory issues, but also issues related to flexibility, attention, and emotion (differences or difficulties in starting, switching, and stopping). Of course, these differences can also bring strengths or joy, such as through stimming (Kapp et al., 2019) or appreciation for sensations such as colors and certain textures. It was a delight to present my related paper (Kapp, 2013) at Jodi's and her conference to further clarify such nuances, and to stand as just one of the countless lives she touched.

Anne Donnellan's expertise and values have proven to be ahead of her time, with science, policy, and practice helping autistic people to have fulfilling lives in the community.

## References

[B1] Carson D. (2017). Walking with Joaquin. Available at: https://www.youtube.com/watch?v=ruXB3lbiD3U (accessed July 24, 2024).

[B2] Cohen C. J. Donnellan A. M. Paul R. (1987). Handbook of Autism and Developmental Disorders. Chichester: Winston.

[B3] Connors J. L. Donnellan A. M. (1993). Citizenship and culture: the role of disabled people in Navajo society. Disabil. Handicap Soc. 8, 265–280.

[B4] Connors J. L. Donnellan A. M. (1998). Walk in beauty: western perspectives on disability and Navajo family/cultural resilience. Resil. Native Am. Immigr. Fam. 2, 159–182.

[B5] Donnellan A. M. (1984). The criterion of the least dangerous assumption. Behav. Disord. 9, 141–150. 10.1177/019874298400900201

[B6] Donnellan A. M. (1999). Invented knowledge and autism: highlighting our strengths and expanding the conversation. J. Assoc. Persons Severe Handic. 24, 230–236. 10.2511/rpsd.24.3.23031806417

[B7] Donnellan A. M. Hill D. A. Leary M. R. (2013). Rethinking autism: implications of sensory and movement differences for understanding and support. Front. Integr. Neurosci. 6:124. 10.3389/fnint.2012.00124 23372546 PMC3556589

[B8] Donnellan A. M. LaVigna G. Negri-Shoultz N. Fassbender L. (1988). Progress Without Punishment: Effective Approaches for Learners With Behavior Problems. New York, NY: Teachers College Press.

[B9] Donnellan A. M. Leary M. R. (1995). Movement Differences and Diversity in Autism/Mental Retardation: Appreciating and Accommodating People with Communication and Behavior Challenges. Madison, WI: DRI Press.

[B10] Donnellan A. M. Leary M. R. Robledo J. (2006). “I can't get started: stress and the role of movement differences for individuals with the autism label,” in Stress and Coping in Autism, eds. G. Baron, J. Groden, G. Groden, and L. Lipsitt (Oxford: Oxford University Press), 205–245.

[B11] Donnellan A. M. Mirenda P. L. Mesaros R. A. Fassbender L. L. (1984). Analyzing the communicative functions of aberrant behavior. J. Assoc. Sever. Handic. 9, 201–212. 10.1177/154079698400900306

[B12] Donnellan A. M. Sabin L. A. Majure L. A. (1992). Facilitated communication: beyond the quandary to the questions. Top. Lang. Disord. 12, 69–82. 10.1097/00011363-199208000-00007

[B13] Donnellan n.d. *Anne M. Donnellan*. Available at: https://loop.frontiersin.org/people/48362/bio (accessed July 24, 2024).

[B14] Donnellan-Walsh A. Gossage L. LaVigna G. W. Schuler A. L. Traphagen J. (1977). Teaching Makes a Difference: A Guide for Developing Successful Programs for Autistic and Other Severely Handicapped Students. Santa Barbara, CA: Santa Barbara County Schools.

[B15] Kapp S. K. (2011). Navajo and autism: the beauty of harmony. Disab. Soc. 26, 583–595. 10.1080/09687599.2011.589192

[B16] Kapp S. K. (2013). Empathizing with sensory and movement differences: moving toward a sensitive understanding of autism. Front. Integr. Neurosci. 7:38. 10.3389/fnint.2013.00038 23745107 PMC3662878

[B17] Kapp S. K. Steward R. Crane L. Elliott D. Elphick C. Pellicano. (2019). ‘People should be allowed to do what they like': autistic adults' views and experiences of stimming. Autism 23, 1782–1792. 10.1177/1362361319829628 30818970 PMC6728747

[B18] LaVigna G. W. Donnellan A. M. (1986). Alternatives to Punishment: Solving Behavior Problems With Non-Aversive Strategies. Dallas, PA: Ardent Media.

[B19] Leary M. R. Donnellan A. M. (2012). Autism: Sensory-Movement Differences and Diversity. Cambridge: Cambridge Book Review Press.

[B20] Robledo J. Donnellan A. M. (2016). Supportive relationships in autism spectrum disorder: perspectives of individuals with ASD and supporters. Behav. Sci. 6:23. 10.3390/bs6040023 27827873 PMC5197936

[B21] Robledo J. Donnellan A. M. Strandt-Conroy K. (2012). An exploration of sensory and movement differences from the perspective of individuals with autism. Front. Integr. Neurosci. 6:107. 10.3389/fnint.2012.00107 23162446 PMC3499780

[B22] Robledo J. A. Donnellan A. M. (2008). Properties of supportive relationships from the perspective of academically successful individuals with autism. Intellect. Dev. Disabil. 46, 299–310. 10.1352/1934-9556(2008)46[299:POSRFT]2.0.CO;2 18671444

[B23] Savarese E. T. Savarese R. J. (2010). Autism and the concept of neurodiversity. Disab. Stud. Quart. 30:43. 10.18061/dsq.v30i1.1061

[B24] Torres E. B. Donnellan A. M. (2015a). Autism: The Movement Perspective. Lausanne: Frontiers Media SA. 10.3389/978-2-88919-509-1 PMC4360560 25852501

[B25] Torres E. B. Donnellan A. M. (2015b). Editorial for research topic “Autism: the movement perspective”. Front. Integr. Neurosci. 9:12. 10.3389/fnint.2015.00012 25852501 PMC4360560

[B26] USD News Center (2008). Summer Autism Conference. University of San Diego. Available at https://www.sandiego.edu/events/detail.php?_focus=30878 (accessed September 5, 2024).

[B27] Wisconsin State Journal. (2024). Anne Mary Donnellan: 1943 to 2024. Available at https://www.legacy.com/us/obituaries/madison/name/anne-donnellan-obituary?id=54902520 (accessed October 12, 2024).

